# Effectivity of benzydamine hydrochloride gargle to reduce propofol consumption in endoscopic retrograde cholangiopancreatography procedure: a randomized controlled trial

**DOI:** 10.1186/s12871-020-00996-x

**Published:** 2020-05-23

**Authors:** Adhrie Sugiarto, Christopher Kapuangan, Aida Rosita Tantri, Vincent Chrisnata

**Affiliations:** Department of Anesthesiology and Intensive Care, Faculty of Medicine, Universitas Indonesia - Cipto Mangunkusumo General Hospital, Pangeran Diponegoro Street No. 71, Jakarta, Indonesia

**Keywords:** Endoscopic retrograde cholangiopancreatography, Propofol, Benzydamine hydrochloride, Sore throat

## Abstract

**Background:**

Endoscopic Retrograde Cholangiopancreatography (ERCP) is a complex endoscopic procedure that requires moderate to deep sedation. Propofol is the sedative agent of choice for sedation in ERCP due to its fast distribution and fast elimination time without a cumulative effect after infusion, resulting in shorter recovery time. Benzydamine hydrochloride is a topical non-steroidal anti-inflammatory drug that has analgesic, local anesthetic, and anti-inflammatory effects that have been known to be effective in reducing postoperative sore throat. Combination of propofol and topical analgesic may provide adequate sedation and reduce propofol consumption. This study aimed to determine the effectivity of benzydamine hydrochloride gargling in reducing propofol consumption in the ERCP procedure.

**Methods:**

This study was a single-blind randomized controlled trial for patients undergoing ERCP procedures at Cipto Mangunkusumo Hospital from August to September 2018. A total of 72 subjects were recruited consecutively and randomly assigned into two groups. The first group received 15 mL of 0.15% benzydamine hydrochloride mouthwash prior to the procedure, whereas the second group received 15 mL of water mouthwash. Additional propofol was administered when patient moved or Ramsay Sedation Scale rose above 4. Cumulative propofol consumption per kg body weight per minute and incidence of postoperative sore throat were recorded in each group. Incidence of desaturation, postoperative nausea vomitting, and dysphagia were also recorded. Data analysis was performed with Statistical Package for the Social Sciences.

**Results:**

Cumulative propofol consumption per minute per kg body weight in the benzydamine hydrochloride group was 152.7 (91.9–238.8) mcg/kg/minute, while in the control group was 200.05 (114.4–380.2) mcg/kg/ minute (*p* = < 0.001). The incidence of sore throat on the 0th, 2nd, and 4th hour for the benzydamine hydrochloride group was 11.4, 11.4, and 5.7%, while in the control group was 50, 52.8, and 36.1% (*p* = < 0.001, < 0.001, 0.003). Desaturation was found in control group whereas none in benzydamine hydrochloride group. Complaints of nausea and vomiting were comparable in both groups.

**Conclusion:**

Benzydamine hydrochloride gargling was effective in reducing cumulative propofol consumption in the ERCP procedure.

**Trial registration:**

Study was registered retrospectively in ClinicalTrials.gov with NCT04167592 on November 8th 2019.

## Background

ERCP is a complex and uncomfortable upper gastrointestinal endoscopic procedure that requires moderate to deep sedation [1]. Propofol is the sedative agent of choice for sedation in ERCP due to its fast distribution and fast elimination time without a cumulative effect after infusion, resulting in shorter recovery time. Propofol may result in hypotension and respiratory depression, especially if used in high doses [2–4]. Topical analgesic is often added to reduce throat irritation incidence which can trigger gag reflex when an endoscopic probe is inserted. Combination of propofol and topical analgesic is an alternative to reduce propofol consumption and to provide adequate sedation while avoiding propofol side effects [3, 5].

Benzydamine hydrochloride is a non-steroidal anti-inflammatory drug that has analgesic, local anesthetic, anti-inflammatory, and anti-microbial effects. The benzydamine hydrochloride is often used as a mouthwash and has been known effective to reduce the incidence of postintubation sore throat in patients undergoing general anesthesia [6]. Research on the effect of benzydamine hydrochloride mouthwash in patients undergoing the ERCP procedure has never been done before. This study aimed to determine the effectivity of benzydamine hydrochloride gargling in reducing propofol consumption in the ERCP procedure.

## Methods

This was a single blind randomized controlled trial aimed to determine the effectiveness of benzydamine hydrochloride mouthwash in reducing propofol requirements and sore throat incidence in ERCP procedure. The study was conducted in Gastrointestinal Endoscopy Center, Cipto Mangunkusumo Hospital, Jakarta - Indonesia from August to September 2018.

The study was registered in ClinicalTrials.gov (NCT04167592) and approved by Ethics Committee in Faculty of Medicine, Universitas Indonesia (0679/UN.2F1/ETIK/2018). The study began after obtaining the subject’s consent.

### Participants

Seventy-two adult subjects undergoing ERCP procedures, aged above 18 years old, ASA I–III, BMI 18–30 kg/m^2^ were consecutively recruited and randomly assigned into two groups. Patients who had history of propofol allergy, had contraindication to propofol administration, had throat wound or laceration, had analgesics or steroids in 24 h prior to the procedure, had unstable hemodynamic condition during sedation that needed changes of sedation technique, had severe vital organ disorder, or had procedure longer than 90 min were excluded from this study.

### Study protocol

Randomization was done by using randomization tool in www.randomizer.org. Concealment were done with closed envelope that was opened pre procedure by a research assistant. Patients were blinded to data collection. Subjects in intervention group gargled with 15 mL of 0.15% benzydamine hydrochloride, while subjects in control group gargled with 15 mL of water three minutes before sedation started. All patients were instructed to spread the mouthwash agent in the mouth, rinsed it for 30 s, and discarded it. Both liquid used the dark-coloured bottle container, and after gargling the patient spat into the black coloured plastic bag. Sedation was started with 1 mcg/kg of fentanyl and 1 mg/kg of propofol administered intravenously. Sedation was maintained with 50 mcg/kg/minute of propofol intravenous infusion. The dosage of propofol intravenous infusion was adjusted according to patient’s Ramsay Sedation Scale (RSS) during procedure. The RSS should be maintained in the scale of 4–5. Patients were given 3 L/minute of O_2_ via nasal canule and they all breathed spontaneously during the procedure. About 0.3 mg/kg of propofol was administered when patients moved or sedation score rose above 4. About 25 mcg of fentanyl was given if heart rate increased 20% above baseline and mean arterial pressure increased more than 20% above baseline. If blood pressure decreased 20% below baseline, 5 mg of ephedrine was given, and propofol intravenous infusion was decreased to 10 mcg/kg. Duration of the procedure began when the endoscopic probe was inserted into the mouth and after the RSS target score of 4–5 was reached.

### Study parameters

This study aimed to determine the effectivity of benzydamine hydrochloride gargling in reducing propofol consumption in ERCP procedure. The primary outcome was the cumulative propofol consumption during ERCP procedure. The secondary outcomes were incidence of sore throat after ERCP procedure. The degree was determined by using a Numeric Rating Scale (NRS) which ranged from 0 to 10, with 0 meaning no pain and 10 meaning very painful. Degree of post sedation sore throat along with incidence of hypotension (a decrease in blood pressure > 20% that was not resolved by vasoconstrictors), desaturation (SpO2 < 90%), nausea, vomiting, and dysphagia were also recorded.

### Sample size calculation

Sample size was calculated according to unpaired numeric analysis and unpaired categorical comparative analysis for multiple measurements. Those calculations were performed according to primary and secondary hypotheses, which were benzydamine effect on reduction in propofol consumption and sore throat incidence, respectively. Based on unpaired numeric analysis for multiple measurements, 30 samples were required. This calculation was based on type I error of 0.05 and type II error of 0.2 whereas standard deviation of 19.23 was used according to prior study. Meanwhile, another sample size calculation according to unpaired categorical comparative analysis for multiple time measurement was performed since the formula was suitable for secondary hypothesis (i.e. calculation of sore throat incidence). The preliminary study regarding this issue has shown that proportion of sore throat found in control group was 0.4 while the significant NRS of sore throat was adjusted as 0.2. Thus, proportion of sore throat in benzydamine hydrochloride group was estimated as 0.2. Type I error of 0.05 and type II error of 0.2 were adjusted with a statistical power of 0.8. In total, the study required 72 subjects with dropout allowance rate of 0.1. Therefore, bigger calculation of minimum sample size of 72 participants were recruited in this study.

### Statistical analysis

All collected data were analyzed with Statistical Package for the Social Sciences version 21.0 (IBM Corp., Armonk, New York, USA). Chi-square test was used to analyze categorical variables while Mann-Whitney test was used to analyze categorical variables as chi-square alternatives and to analyze numerical variables for abnormal distribution data. Unpaired t-test was used to analyze numerical variables for normal distribution data. Normal distribution of numerical variables was displayed in mean (SD) whereas abnormal distribution was displayed in median with minimum to maximum values. Categorical variables were displayed in percentage. *P*-value less than 0.05 was stated as significant.

## Results

**Fig. 1:**
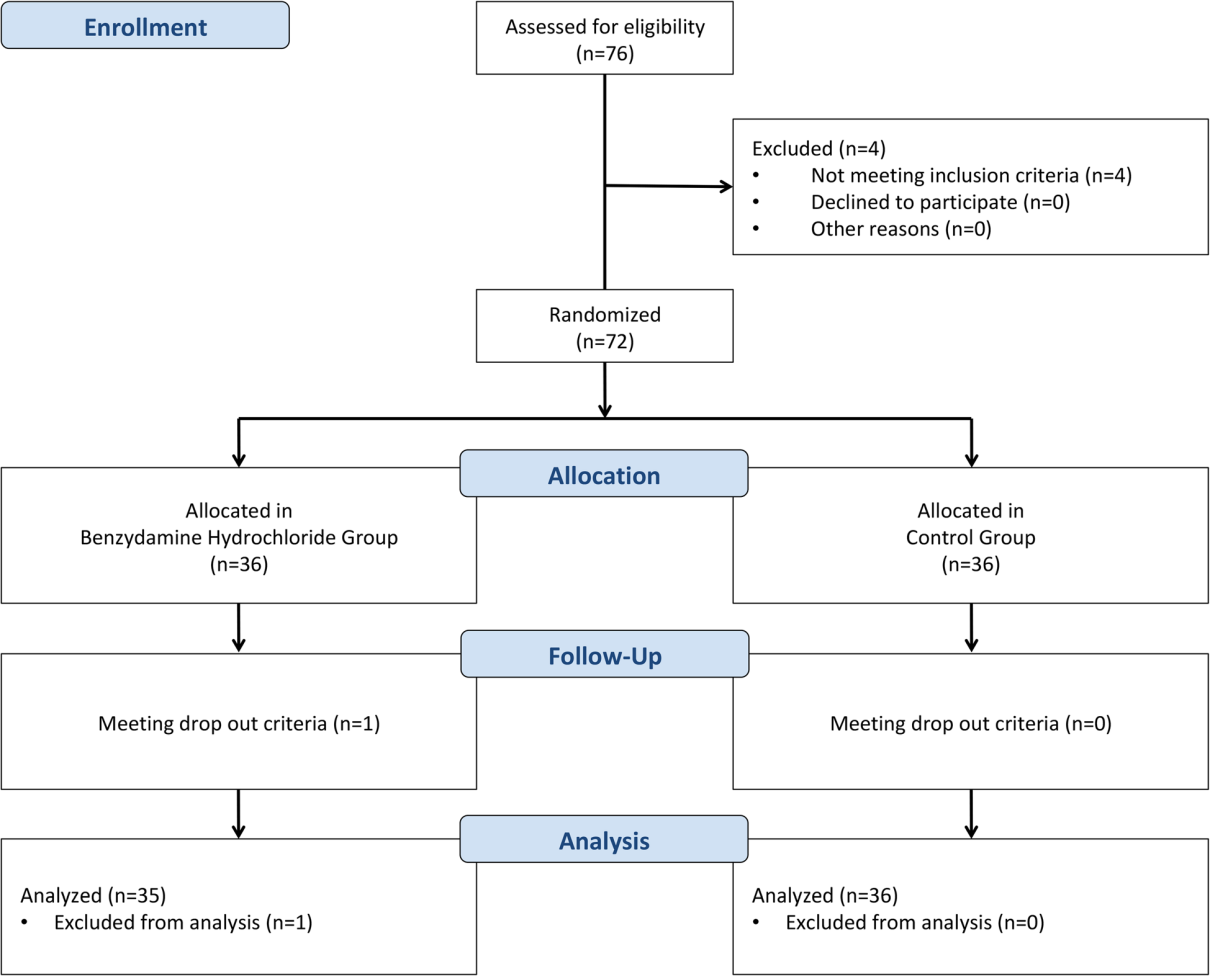
Flowchart of subjects’ recruitment process

72 subjects were recruited from August to September 2018 in this study, one subject in benzydamine group was excluded due to unstable condition [Fig. 1]. Because one subject was excluded, only 35 subjects remained in benzydamine hydrochloride group and was put into analyses. The description of subjects’ characteristics is shown in Table 1.

**Table 1:** Characteristics of Research Subjects

Variable	Control Group ***n*** = 36	Benzydamine Hydrochloride Gargle Group ***n*** = 35	***p***
**Sex**			
Male (%)	44.4	42.9	0.893^b^
Female (%)	55.6	57.1	
**Age (years)**	48.97 ± 11.21	46.74 ± 12.64	0.434^a^
**ASA Status**			
ASA 1 (%)	8.3	2.9	0.349^c^
ASA 2 (%)	72.2	71.4	
ASA 3 (%)	19.4	25.7	
**Body Weight (kg)**	58 (42–98)	54 (39–90)	0.322^c^
**BMI (kg/m**^**2**^**)**	22.5 (18–29.8)	22.2 (18–29.8)	0.272^c^
**Duration of procedure (minute)**	35 (15–89)	46 (15–90)	0.210^c^
**Total fentanyl requirement (mcg)**	75 (50–200)	75 (50–100)	< 0.614^c^

^a^ Unpaired T-Test. ^b^ Chi Square Test

^c^ Mann-Whitney Test

Cumulative propofol consumption per minute per kilogram body weight was the amount of propofol consumption during the procedure (mcg) divided by time (minutes) and body weight (kg). Cumulative propofol consumption in benzydamine group was significantly different than in control group (Table 2).

**Table 2:** Cumulative Propofol Consumption and Sore Throat Incidence

	Time	Control Group ***n*** = 36	Benzydamine Hydrochloride Gargle Group ***n*** = 35	***p***
**Cumulative propofol consumption (mcg/kg/minute)**		200.05 (114.4–380.2)	152.7 (91.9–238.8)	< 0.001^a^
**Sore throat incidence (%)**	0th Hour	50.00.00	11.04	< 0.001^b^
	2nd Hour	52.08.00	11.04	< 0.001^b^
	4th Hour	36.01.00	05.07	0.003^b^

^a^Mann-Whitney Test

^b^Chi Square Test

In this study, the assessment of sore throat was done at three different times; at the 0th hour when the patient in fully awake condition (Alderette Score of 10), the 2nd hour, and the 4th hour postsedation. The postsedation sore throat incidence between two groups were significantly different (Table 2, *p* < 0.001, *p* < 0.001, *p* = 0.003 respectively).

In this study, an assessment for the degree of postsedation sore throat was also carried out at three different times; at the 0th hour when the patient was fully awake (Alderette Score of 10), the 2nd hour, and the 4th hour post-action. The difference for degree of sore throat between the two groups at 0th, 2nd, and 4th hours is presented in Table 3.

**Table 3:** Degree of Postsedation Sore Throat

Time	Control Group (%)				Benzydamine Hydrochloride Gargle Group (%)				***p***
	0	1	2	3	0	1	2	3	
0th Hour	50.0	36.1	11.1	2.8	88.6	11.4	0	0	0.001^a^
2nd Hour	47.2	47.2	5.6	0	88.6	11.4	0	0	< 0.001^a^
4th Hour	63.9	33.3	2.8	0	94.3	5.7	0	0	0.002^a^

^a^Mann Whitney Test

The occurrence of side effects in the form of hypotension, desaturation, nausea vomiting and dysphagia were recorded in both groups and can be seen in Table 4.

**Table 4:** Incidence of Hypotension, Desaturation, Nausea, Vomiting, and Dysphagia

Variable	Control Group ***n*** = 36	Benzydamine Hydrochloride Gargle Group ***n*** = 35
Hypotension	3 (8.3%)	2 (5.7%)
Desaturation	3 (8.3%)	0
Nausea Vomiting	5 (14.3%)	5 (16.1%)
Dysphagia	0	0

Value expressed in n (%)

## Discussion

This was the first study using benzydamine hydrochloride mouthwash prior to ERCP procedure. The results showed that cumulative propofol requirements in the benzydamine hydrochloride group was significantly less than in the control group. The result is consistent with other studies using topical anaesthetics in endoscopic procedures conducted by Soweid et al. (2011) and Basturk et al. (2017) which used lidocaine in the form of spray or gel [3, 5]. However, lidocaine spray might cause irritation, nausea, vomiting, and dysphagia. Thus, it was not commonly used and benzydamine hydrochloride mouthwash became an alternative for this approach.

Benzydamine hydrochloride has been used in years to reduce the mucosal inflammation due to radiotherapy radiation and to reduce the incidence of sore throat postprocedure in patients undergoing general anaesthesia with endotracheal intubation or laryngeal mask airway devices [7, 8]. The agent was used as a mouthwash before the procedure began. Gargling is effective in distributing the agent into oropharyngeal area, posterior pharyngeal wall, anterior epiglottis area, and uvula [8, 9]. Thus, those areas would be covered by the agent and it reduced the sore throat in patients undergoing the procedure.

Sore throats are usually complained after the procedure due to inflammation on the mucosa. This inflammation will improve after some time, as the literature says it will improve in the first 24–72 h postoperatively [10]. However, this study found that pain caused by sore throat could be reduced until 4 h postsedation with the use of benzydamine hydrochloride mouthwash before the procedure. The agent might give topical anaesthesia effect until 90 min after administration. Moreover, the complaint of sore throat was significantly reduced due to anti-inflammatory effect from the agent.

Benzydamine hydrochloride has several common features to other nonsteroidal anti-inflammatory drugs (NSAIDs) such as analgesia effect and anti-inflammatory effect. The local analgesia effect worked only on localized inflammatory factors without any interaction to systemic mechanism [9]. Moreover, the anti-inflammatory effect might occur due to its ability in modulating the transformation of blood vessels and in suppressing the work of phagocytic cells. Therefore, the occurrence of vasodilation and the increase of vascular permeability would be prevented while the release of granules and lytic enzymes would be reduced [9, 11].

The agent is recommended to be given about 4 mmol/L in 15 mL and gargled for 30 s. The agent should be administered in high concentration because only small amount of this agent would work effectively while the rest of it would be dissolved in saliva. The diffusion depth of topical administration is not exactly understood. However, some studies stated that most of it would be more concentrated in the tissue surface compared to when administered systemically [8, 9]. The local anesthesia effect of benzydamine hydrochloride might be the cause of significant decrease of cumulative propofol consumption in this study.

At concentration of 10–100 μmol/L, benzydamine hydrochloride may stabilize the mucosal membrane whereas at concentration of 3–30 μmol/L, it might inhibit the release of azurophilic granules from neutrophils. Similar mechanism has been shown by other drugs which have membrane stabilization effects such as beta blockers, local anesthetics, and some NSAIDs with acidic features when given in high doses. In experiment involving fat cells in vitro, benzydamine hydrochloride might increase the formation of *cyclic* 3′,5′-AMP which affects the activity of intracellular cation. This effect may originate from the activity of local anesthetics held by benzydamine hydrochloride [8].

In this study, desaturation occurred in the control group while hypotension occurred in both groups. These side effects might be related to propofol usage during the ERCP procedure and were not related to benzydamine hydrochloride. Propofol has been known to induce desaturation, hypotension, apnea, allergic reactions, and cardiac arrest [12, 13]. However, the incidence of desaturation and hypotension was higher in the control group than in benzydamine hydrochloride group. This could be caused by higher cumulative dose for propofol in the control group. Hence, the incidence of hypotension and desaturation will increase in proportion to the increase of propofol dose [14].

Other minor side effects that can happen during the use of benzydamine hydrochloride are nausea, vomiting, and dysphagia due to numbness in the mouth [9]. In this study, there was no incidence of dysphagia in either control group or the benzydamine hydrochloride group while complaints of nausea and vomiting were comparable in both groups. However, the ERCP procedure itself can also precipitate nausea and vomiting due to contrast used during procedure or pancreatic inflammation, which is a serious complication of this procedure [15, 16]. In addition, the use of fentanyl in ERCP anesthesia procedure may cause nausea and vomiting postprocedure [12].

The single use of Ramsay Sedation Scale, as an indicator to assess the depth of sedation, was the limitation in this study. This scale might lead to bias because the depth of anesthesia can be more objective if assessed with Bispectral Index (BIS). The use of BIS monitors has been known to provide anesthesiologists in assessing the depth of anesthesia in patients receiving sedation [17]. Furthermore, other possible bias was the taste and appearance of water, compared to benzydamine hydrochloride. In order to maintain blindness for the patient, dark colored container was used and it was discarded into black colored plastic bag. There was no explanation regarding the taste to the patient prior to the procedure. Moreover, there was no contact between each patient from each group before and after the procedure. In this study, endoscopist’s statisfaction towards this method has not been further elaborated. However, this might lead to a subjective satisfaction ratings according to different perspectives.

## Conclusion

Benzydamine hydrochloride gargle is effective in reducing cumulative propofol consumption during ERCP procedure.

## Data Availability

All data generated or analyzed during this study are presented in this manuscript and/or additional supporting files. The additional datasets are also available from the corresponding author on reasonable request.

## Abbreviations

AMP: Adenosine monophosphate
ASA: American Society of Anesthesiologists classification
BMI: Body mass index
ERCP: Endoscopic retrograde cholangiopancreatography
NRS: Numerical rating scale
NSAIDs: Nonsteroidal anti-inflammatory drug
RSS: Ramsay sedation scale
BIS: Bispectral index
